# The Genes Coding for the Conversion of Carbazole to Catechol Are Flanked by IS*6100* Elements in *Sphingomonas* sp. Strain XLDN2-5

**DOI:** 10.1371/journal.pone.0010018

**Published:** 2010-04-02

**Authors:** Zhonghui Gai, Xiaoyu Wang, Xiaorui Liu, Cui Tai, Hongzhi Tang, Xiaofei He, Geng Wu, Zixin Deng, Ping Xu

**Affiliations:** MOE Key Laboratory of Microbial Metabolism and School of Life Sciences and Biotechnology, Shanghai Jiao Tong University, Shanghai, People's Republic of China; BMSI-A*STAR, Singapore

## Abstract

**Background:**

Carbazole is a recalcitrant compound with a dioxin-like structure and possesses mutagenic and toxic activities. Bacteria respond to a xenobiotic by recruiting exogenous genes to establish a pathway to degrade the xenobiotic, which is necessary for their adaptation and survival. Usually, this process is mediated by mobile genetic elements such as plasmids, transposons, and insertion sequences.

**Findings:**

The genes encoding the enzymes responsible for the degradation of carbazole to catechol via anthranilate were cloned, sequenced, and characterized from a carbazole-degrading *Sphingomonas* sp. strain XLDN2-5. The *car* gene cluster (*carRAaBaBbCAc*) and *fdr* gene were accompanied on both sides by two copies of IS*6100* elements, and organized as IS*6100*::IS*Ssp1*-*ORF1-carRAaBaBbCAc-ORF8*-IS*6100*-*fdr*-IS*6100*. Carbazole was converted by carbazole 1,9a-dioxygenase (CARDO, CarAaAcFdr), *meta*-cleavage enzyme (CarBaBb), and hydrolase (CarC) to anthranilate and 2-hydroxypenta-2,4-dienoate. The *fdr* gene encoded a novel ferredoxin reductase whose absence resulted in lower transformation activity of carbazole by CarAa and CarAc. The *ant* gene cluster (*antRAcAdAbAa*) which was involved in the conversion of anthranilate to catechol was also sandwiched between two IS*6100* elements as IS*6100*-*antRAcAdAbAa*-IS*6100*. Anthranilate 1,2-dioxygenase (ANTDO) was composed of a reductase (AntAa), a ferredoxin (AntAb), and a two-subunit terminal oxygenase (AntAcAd). Reverse transcription-PCR results suggested that *carAaBaBbCAc* gene cluster, *fdr*, and *antRAcAdAbAa* gene cluster were induced when strain XLDN2-5 was exposed to carbazole. Expression of both CARDO and ANTDO in *Escherichia coli* required the presence of the natural reductases for full enzymatic activity.

**Conclusions/Significance:**

We predict that IS*6100* might play an important role in the establishment of carbazole-degrading pathway, which endows the host to adapt to novel compounds in the environment. The organization of the *car* and *ant* genes in strain XLDN2-5 was unique, which showed strong evolutionary trail of gene recruitment mediated by IS*6100* and presented a remarkable example of rearrangements and pathway establishments.

## Introduction

**Figure 1:**
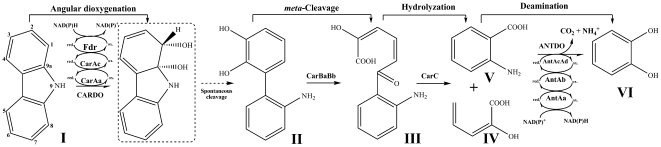
Biodegradation of carbazole via the angular pathway by *Sphingomonas* sp. XLDN2-5 and other carbazole-utilizing bacteria. The product in dashed square is unstable and has not been detected. Enzyme names: carbazole 1,9a-dioxygenase (*carAaAcfdr*); *meta*-cleavage enzyme (*carBaBb*); hydrolase (*carC*); anthranilate 1,2-dioxygenase (*antAcAdAbAa*). Compound I: carbazole; compound II: 2′-aminobiphenyl-2,3-diol; compound III: 2-hydroxy-6-oxo-6-(2′-aminobiphenyl)-hexa-2,4-dienoic acid; compound IV: 2-hydroxypenta-2,4-dienoate; compound V: anthranilic acid; VI: catechol.

Carbazole is an *N*-heterocyclic compound that is known to possess mutagenic and toxic activities [1]. To date, a number of bacterial strains capable of degrading carbazole have been isolated and characterized. Phylogenetically, almost all of these strains belong to pseudomonads and sphingomonads. The degradation of carbazole starts with angular dioxygenation in all studied strains so far [2], [3], [4], [5], [6], [7], [8]. In this pathway, carbazole is initially attacked at the 1 and 9a positions by carbazole 1,9a-dioxygenase (CARDO), resulting in the formation of a highly unstable hemiaminal, which gives rise to 2′-aminobiphenyl-2,3-diol after spontaneous cleavage and rearomatization. An extradiol dioxygenase attacks the hydroxylated ring of 2′-aminobiphenyl-2,3-diol at the *meta* position to generate 2-hydroxy-6-(2′-aminophenyl)-6-oxo-2,4-hexadienoic acid, which is hydrolyzed to produce anthranilic acid and 2-hydroxypenta-2,4-dienoic acid. The resulting metabolite, anthranilate, is converted to catechol in a single step by anthranilate 1,2-dioxygenase (ANTDO) (Figure 1) [9], [10]. In this upper carbazole degradation pathway, CARDO is considered as the key enzyme. In *Pseudomonas resinovorans* CA10, the most intensively studied pseudomonad, CARDO consists of a terminal oxygenase, a ferredoxin, and a ferredoxin reductase, encoded by *carAa*, *carAc*, and *carAd*, respectively. In pseudomonads, the upper pathway genes, *carAaBaBbCAcAd*, are in the *car* cluster, which is transcribed as a single transcriptional unit [6]. Interestingly, unlike the well-organized operons in pseudomonads, the catabolic genes in sphingomonads are often dispersed or not coordinately regulated. For example, plasmid pCAR3 contains multiple gene sets, which are involved in the carbazole degradation pathway in a carbazole-degrader *Novosphingobium* sp. KA1 (previous *Sphingomonas* sp. KA1) [10]. These unusual organizations of the degradative genes were often observed in other sphingomonads [11], [12].

Insertion sequences (ISs) are a subject of increasing interests for biodegradation because of the variety of their structures, modes of action, and the biodegradation abilities they confer bacteria [13]. ISs are small and mobile genetic elements that are ubiquitously distributed within bacterial genomes, and play an important role in evolution by facilitating horizontal gene transfers between bacterial populations, which contribute significantly to the diversity of bacteria by enhancing the organisms' adaptive and evolutionary capacities. IS*6100* is an important IS that flanks a range of catabolic operons, for example, the operons for metabolism of various aromatic substrates [14], [15]. In this work, we report that two loci coding for the enzymes that convert carbazole to catechol were found to be flanked by IS*6100* elements. Evidence was given for the involvement of these genes in the degradation of carbazole in *Sphingomonas* sp. XLDN2-5.

## Results

### Screening of the genomic library, DNA sequencing, and genome walking

**Figure 2:**
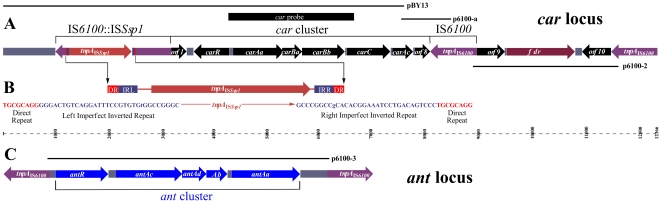
Physical maps of *car* and *ant* loci. (**A**) Physical map of *car* locus, which is delimited by IS*6100* elements. The upstream IS*6100* is interrupted by a novel insert element IS*Ssp1*, and was designated IS*6100*::IS*Ssp1*. (**B**) Schematic representation of the main features of the novel IS*Ssp1* sequence in *Sphingomonas* sp. XLDN2-5. The orientation of the IS*Ssp1* is shown by an arrow. The red and blue boxes represent the positions of two direct repeats (DR) and two imperfect, 32 bp, terminal inverted repeats (the left inverted repeat [IRL], and the right inverted repeat [IRR]) with one mismatch, which is indicated by lowercase letters. The nucleotide sequences of DRs and IRs are also given. (**C**) Physical map of *ant* cluster which is franked by IS*6100* elements along a base pair scale.

The *car* probe, labeled with DIG, was used for screening the genomic library, and a positive clone, designated as pBY13 (Figure 2A), was sequenced and analyzed to contain a DNA insert of 6.8 kb. BLAST search results revealed that there were five intact open reading frames (ORFs), *carR*, *carAa*, *carBa*, *carBb*, and *carC*, which were found to be 99% identical to the corresponding genes of *Novosphingobium* sp. strain KA1 [10]. A closer look at the left region suggested that an IS*6100*, exhibiting 100% identity to that of *Mycobacterium fortuitum* [16], was interrupted by a novel insertion element IS*Ssp1*. Therefore, the interrupted IS*6100* was designated IS*6100*::IS*Ssp1*. The putative transposase for IS*Ssp1* was transcribed in the same direction of the *car* cluster, but in the opposite direction as the interrupted IS*6100*. IS*Ssp1* belongs to the IS*256* family of prokaryotic ISs, a subgroup of the mutator family of transposases (Pfam00872). IS*Ssp1* which had imperfect inverted repeats (32-bp in length, one mismatch) was flanked by two copies of 8-bp direct repeat (Figure 2B).

At the 3′-end of pBY13, an incomplete ORF (238 bp) showed 100% identity to *carAcI* of KA1. In order to clone the full gene and its flanking sequences, a 1-kb DNA was amplified by genome walking. After ligation of the fragment to pMD18-T followed by transformation to *E. coli* DH5α, a positive clone, designated p6100-a, was obtained. DNA sequencing indicated that p6100-a contained the left part of *carAc*, an ORF8, and interestingly a partial IS*6100* sequence. The presence of the partial IS*6100* sequence led to the hypothesis that there was a complete IS*6100* and might form a transposon, and therefore triggered further study. The presence of an intact IS*6100* copy downstream of ORF8 was successfully validated by PCR using carC-sp3 and IS6100-F2 and sequencing. This transposon-like context (IS*6100*::IS*Ssp1*-*ORF1-carRAaBaBbCAc-ORF8*-IS*6100*) was designated Tn*Car*, tentatively.

### IS-based PCR

The presence of IS*6100* on both sides of the *car* gene cluster motivated further investigations using IS-based PCR. An IS-based PCR with primers IS6100-F1 and IS6100-R1 targeting the IS*6100* element resulted in two distinct PCR products of about 4.3 and 2.8 kb, respectively (Figure S1B, lane 1). The 4.3 and 2.8-kb fragments were ligated to pMD18-T to generate plasmid p6100-1 and p6100-2. DNA sequencing and BLAST search suggested that there were two partial IS*6100* at each end as expected in the 4.3 and 2.8-kb fragments. The 4.3-kb fragment contains *ORF1carRAaBaBbCAcORF8* from Tn*Car*.

BLAST search suggested that in the 2.8-kb fragment, there were two truncated ORFs and one intact ORF (Fdr) that showed 62% identity to the FdrI of strain KA1 [10]. A sequence comparison showed that the amino acid sequence of Fdr was homologous over its entire length to other members of the FAD-dependent pyridine nucleotide reductase family, containing a flavin binding domain for FAD (consensus sequence T*X*_6_A*X*GD) and two ADP binding domains (for FAD and NADH, respectively) with the consensus sequence G*X*G*X*_2_G*X*_3_A [17], [18], [19]. Two truncated ORFs encoded for a putative uncharacterized protein and a transcriptional regulator, were found upstream and downstream of *fdr* (Table 1). Sequencing of PCR product revealed the existence of entire copies of IS*6100* at both ends of the 2.8-kb fragment. These two IS*6100* were in the same direction and formed a composite transposon (IS*6100*-*fdr*-IS*6100*) designated Tn*Fdr*, tentatively.

**Table 1:** Coding regions of *car* locus and *ant* locus.

Protein	Position (bp) in sequence (direction)	Length (amino acids)	Putative function	Homologous protein		
				% Identity	Protein (accession no.)	Source
***car*** ** locus**						
TnpA_IS*6100*_	1116–1263; 2594–3240 (c)	264	Transposase of IS*6100*	100	Tnp (Q79AS6)	*Mycobacterium fortuitum*
TnpA_IS*Ssp1*_	1321–2544 (n)	407	Transposase, mutator type	99	Tnp (A5VHF2)	*Sphingomonas wittichii* RW1
ORF1	3272–3400 (n)	42	Integrase family protein (truncated, only C-terminal portion)	100	ORF7 (Q84IH1)	*Novosphingobium* sp. KA1
CarR	3621–4301 (c)	226	Transcriptional regulator of car operon, GntR family	99	CarR (Q84IH0)	*Novosphingobium* sp. KA1
CarAa	4404–5540 (n)	378	Terminal oxygenase component of carbazole 1,9a-dioxygenase	99	CarAaI (Q84IG9)	*Novosphingobium* sp. KA1
				60	CarAa (Q8G8B6)	*Pseudomonas resinovorans* CA10
CarBa	5489–5821 (n)	110	small subunit of *meta* cleavage enzyme	100	CarBaI (Q84IG8)	*Novosphingobium* sp. KA1
CarBb	5814–6617 (n)	267	large subunit of *meta* cleavage enzyme	99	CarBbI (Q84IG7)	*Novosphingobium* sp. KA1
				42	CarBb (Q4TTW1)	*Pseudomonas* sp. XLDN4-9
CarC	6660–7484 (n)	274	Meta cleavage compound hydrolase	99	CarCI (Q84IG6)	*Novosphingobium* sp. KA1
CarAc	7525–7854 (n)	109	Ferredoxin component of carbazole 1,9a-dioxygenase	100	CarAcI (Q84IG5)	*Novosphingobium* sp. KA1
				57	CarAcII (Q2PFA2)	*Novosphingobium* sp. KA1
ORF8	7896–8117 (n)	74	TonB-dependent receptor (truncated, only N-terminal portion)	100	ORF35 (Q84IG4)	*Novosphingobium* sp. KA1
TnpA_IS*6100*_	8172–8966 (c)	264	Transposase of IS*6100*	100	Tnp (Q79AS6)	*Mycobacterium fortuitum*
ORF9	8998–9545 (n)	182	Putative uncharacterized protein (truncated, only C-terminal portion)	34	Q74F08	*Geobacter sulfurreducens*
Fdr	9573–10817 (n)	407	Ferredoxin reductase component of carbazole 1,9a-dioxygenase	62	FdrI (Q2PF96)	*Novosphingobium* sp. KA1
				59	FdrII (Q2PF93)	*Novosphingobium* sp. KA1
ORF10	10936–11486 (c)	182	Transcriptional regulator, TetR family (truncated, only C-terminal portion)	40	Q1NF20	*Sphingomonas* sp. SKA58
TnpA_IS*6100*_	11541–12335 (c)	264	Transposase of IS*6100*	100	Tnp (Q79AS6)	*Mycobacterium fortuitum*
***ant locus***						
TnpA_IS*6100*_	55–849 (c)	264	Transposase of IS*6100*	100	Tnp (Q79AS6)	*Mycobacterium fortuitum*
AntR	1009–1965 (n)	318	Transcriptional regulator, AraC family	100	AndR (Q0KJU2)	*Novosphingobium* sp. KA1
				41	AndR (Q84BZ4)	*Burkholderia cepacia* DPO1
AntAc	2163–3446 (n)	427	Anthranilate 1,2-dioxygenase large subunit	100	AndAc (Q0KJU3)	*Novosphingobium* sp. KA1
				76	AndAc (Q84BZ3)	*Burkholderia cepacia* DPO1
AntAd	3451–3921 (n)	156	Anthranilate 1,2-dioxygenase small subunit	100	AndAd (Q0KJU4)	*Novosphingobium* sp. KA1
				59	AndAd (Q84BZ2)	*Burkholderia cepacia* DPO1
AntAb	3935–4243 (n)	102	Ferredoxin component of anthranilate 1,2-dioxygenase	100	AndAb (Q0KJU5)	*Novosphingobium* sp. KA1
				53	AndAb (Q84BZ1)	*Burkholderia cepacia* DPO1
AntAa	4364–5605 (n)	413	Ferredoxin reductase component of anthranilate 1,2-dioxygenase	100	AndAa (Q0KJU6)	*Novosphingobium* sp. KA1
				40	AndAa (Q84BZ0)	*Burkholderia cepacia* DPO1
TnpA_IS*6100*_	6183–6977 (n)	264	Transposase of IS*6100*	100	Tnp (Q79AS6)	*Mycobacterium fortuitum*

Tn*Car* and Tn*Fdr* were each flanked by two copies of IS*6100* in tail-to-head configuration. Although tail-to-head was the most abundant case for two copies of IS*6100*, other configurations were also reported [14]. Early attempts to amplify head-to-head and tail-to-tail configurations proved to be unsuccessful. Considering that the amplification of head-to-head and tail-to-tail configurations may be suppressed by intramolecular hybridization between two IS*6100* copies during the primer annealing phase of PCR, TaKaRa LA Taq and GC Buffer I were used in the following experiments. One specific fragment (5.2 kb in size, Figure S1C, lane 3) was amplified using IS6100-R1, while no specific fragment was amplified under the same PCR conditions using IS6100-F1 (Figure S1C, lane 2). The 5.2-kb fragment was gel-purified and cloned to pMD18-T to generate p6100-3, and the nucleotide sequence of the 5.2 kb insert was determined. In the sequenced region, five intact ORFs were found to be almost identical (only one bp mismatch) to *andRAcAdAbAa* genes of KA1 whose products were expected for catalyzing the conversion of anthranilate to catechol. The five ORFs were designated *antR*, *antAc*, *antAd*, *antAb*, and *antAa* (Figure 2C). Strain XLDN2-5 ANTDO was a three-component dioxygenase and composed of a two-subunit oxygenase (*antAcAd*), a Rieske-type ferredoxin (*antAb*), and a ferredoxin reductase (*antAa*). *antR*, encoding a putative transcriptional activator, was in the same direction of *antAcAdAbAa*, which was different from that of *Burkholderia cepacia* DBO1 whose *antR* is in the opposite direction to its structure genes [20]. In the sequenced region, there were two partial IS*6100* at each end as expected. The existence of intact IS*6100* elements at both ends was confirmed by PCR and PCR product sequencing. These two IS*6100* elements were in the head-to-head configuration and formed a composite transposon designated Tn*Ant*, tentatively.

### The positional relation of Tn*Car,* Tn*Fdr* and Tn*Ant*

There were two copies of IS*6100* elements at both ends of the three transposon-like entities except that the upstream flanking IS*6100* on Tn*Car* was disrupted by a novel insertion sequence IS*Ssp1*. It was likely that one IS*6100* element was shared by two transposon-like units. In order to analyze the positional relation of Tn*Car*, Tn*Fdr*, and Tn*Ant*, five primers were designed outside of the IS*6100* elements (Figure S2A). If there was a shared IS*6100* element, a 0.9-kb DNA fragment containing IS*6100* could be amplified by PCR using different primer pairs. As shown in Figure S2B, the expected 0.9-kb fragment could only be amplified using primers TnCar-F1 and TnFdr-R1. These results suggested that Tn*Fdr* shared an IS*6100* element with Tn*Car*, but not with Tn*Ant*. The conclusion was confirmed by second-round PCR (Figure S2C and S2D). Thus, we renamed Tn*Car* and Tn*Fdr* to the *car* locus, and Tn*Ant* to the *ant* locus.

### Description of *car* and *ant* loci

The structures of *car* locus and *ant* locus are depicted in Figure 2 and the ORFs are given in Table 1. Both *car* cluster and *fdr* gene were sandwiched between two copies of IS*6100*. Interestingly, two identical copies of the IS*6100* element also flanked the *ant* gene cluster, possibly making a composite transposon. All five sequenced copies of IS*6100* from strain XLDN2-5 were identical over the entire 880 bp. Direct repeats (TGCGCAGG) were found directly upstream and downstream of IS*Ssp1*, whereas no direct repeat was found outside of the IS*6100* box. Furthermore, the IS*Ssp1* consisted of inverted repeats of 32 bp, of which only one base pair was not identical, and a 1224-bp ORF (tnpA_IS*Ssp1*_), encoding a 407 aa putative transposase that showed similarities to transposases of the IS*256* family. In order to illustrate that the genes on these two loci were really working in the degradation of carbazole in strain XLDN2-5, transcriptional and functional analyses were performed.

### Transcriptional analyses of *car* and *ant* genes

**Figure 3:**
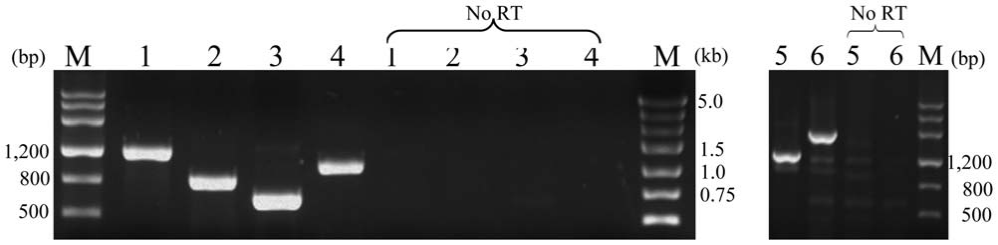
Electrophoresis results of RT-PCR. *carAaBaBb* (lane 1), *carBbC* (lane 2), *carCAc* (lane 3), *fdr* (lane 4), *antAa* (lane 5) and *antAcAdAb* (lane 6). Samples containing no reverse transcriptase (No RT) are also shown.

The expression of the genes presenting on the two loci was studied by reverse transcription (RT)-PCR experiments. The primer sets for the carAaBaBb*CAc*, *fdr*, and *antAcAdAbAa* genes could amplify DNA fragments with the expected sizes (Figure 3). No fragments could be amplified using RNA from glucose-grown XLDN2-5 cells as a template (data not shown). These results revealed that *carAaBaBbCAc*, *fdr*, and *antAcAdAbAa* genes were expressed in carbazole grown XLDN2-5 cells, suggesting that the gene products should be involved in the transformation of carbazole to catechol. These results also indicated that *carAaBaBbCAc* and *antAcAdAb* gene clusters were operonic. In order to confirm that Fdr was active in the CARDO system, functional analyses were performed.

### Functional analyses of putative CARDO and ANTDO

**Figure 4:**
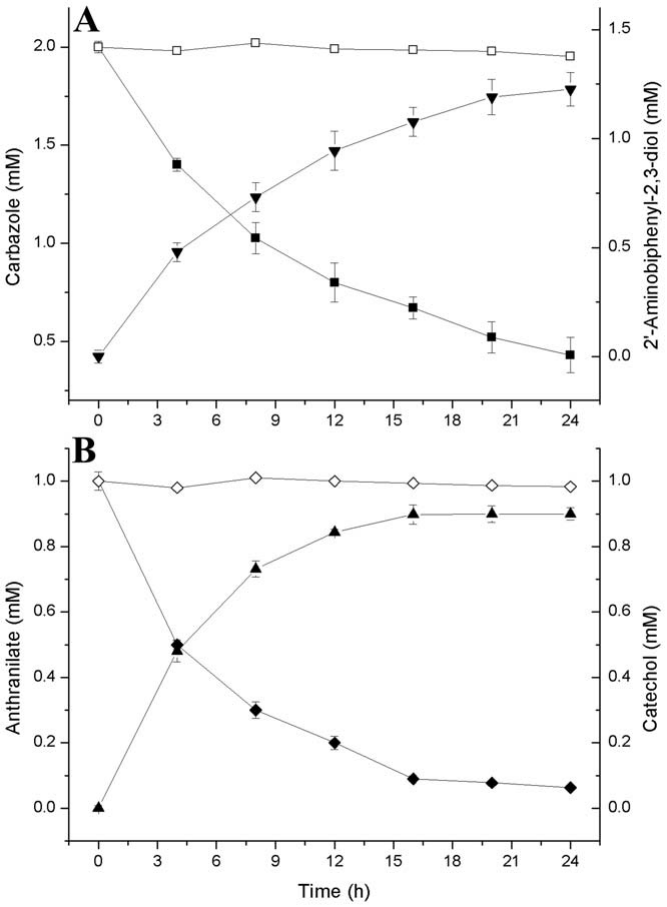
Biotransformation of substrates and accumulation of products. (**A**) Biotransformation of carbazole (-▪-) and accumulation of 2′-aminobiphenyl-2,3-diol (-▾-) by *E*. *coli* DH5α harboring pUcarAaAcfdr. (**B**) Biotransformation of anthranilate (-♦-) and accumulation of catechol (-▴-) by *E*. *coli* DH5α harboring pUantAcdba. *E*. *coli* DH5α harboring pUC19 (-□- and -◊-) served as controls. The initial concentrations of carbazole and anthranilate were 2 mM and 1 mM, respectively. Values are means of three replicates ± SD.

Biotransformation experiments were carried out using *E. coli* cells expressing putative CARDO components (CarAa, CarAc and Fdr) and ANTDO components (AntAc, AntAd, and AntAaAb) (Figure 4). 2′-Aminobiphenyl-2,3-diol and hydroxycarbazole could be detected for incubations of cell harboring pUcarAaAcfdr (Figure S3). About 80% carbazole was converted to 2′-aminobiphenyl-2,3-diol (Figure 4A). These results clearly indicated that CarAa could catalyze angular dioxygenation and lateral dioxygenation of carbazole using CarAc and Fdr as an electron-transfer system. *E. coli* DH5α (pUcarAaAc) encoding only the oxygenase and ferredoxin components transformed no more than 0.1% of the carbazole, which suggested that CarAc had the ability to accept electrons from unidentified reductase in *E*. *coli*, with a much lower efficiency than the natural reductase. Furthermore, CarAc showed similarity with the cytochrome *P*_450_-type reductase component (RedA2) of dioxin dioxygenase from *Sphingomonas wittichii* RW1 [17] and putidaredoxin-type ferredoxins (CamA) from *Pseudomonas putida* [19]. CARDO systems of strain XLDN2-5 can be classified in the class IIA Rieske non-heme iron oxygenase system [10].

The IPTG-induced *E*. *coli* cells carrying pUantAcdba or pUantAcdb readily converted anthranilate to catechol. *E*. *coli* DH5α (pUAntAcdba) completely transformed 1 mM anthranilate to catechol in 24 h (Figure 4B), while *E*. *coli* DH5α (pUAntAcdb) encoding only ferredoxin and oxygenase transformed only 5% of the anthranilate to catechol (data not shown). These results suggested that the expression of CARDO and ANTDO in *E. coli* requires the presence of the ancillary reductases for full enzymatic activity.

## Discussion

Starting from the *carAaBaBb* fragment isolated from *Sphingomonas* sp. strain XLDN2-5, a combination of southern blot, genome walking, and IS-based PCR led to the isolation of two loci containing genes involved in the conversion of carbazole from anthranilate to catechol. The DNA fragments obtained are illustrated in Figure 2, and the proposed reaction details are given in Figure 1. All genes were embedded in transposon-like entities, implying the likely involvement of horizontal gene transfer in the evolution of carbazole degradation pathway. Transcriptional and functional analyses suggested that all the genes worked in the degradation of carbazole. The CARDO system catalyzed angular and lateral dioxygenation of carbazole, and ANTDO could transform anthranilate to catechol in a single step. Our research provided useful and important information for the association of IS*6100* element with carbazole and anthranilate catabolic genes.

**Figure 5:**
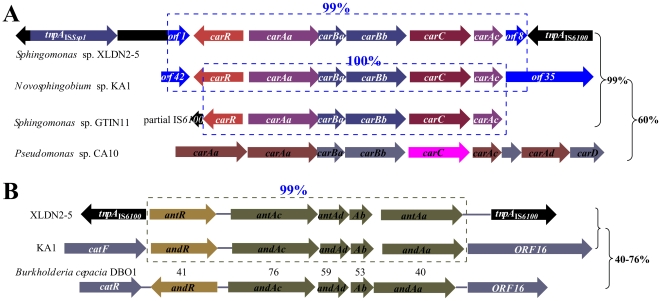
Comparative analyses of *car* and *ant* genes from *Sphingomonas* sp. XLDN2-5 and other strains. (**A**) Comparative analysis of *car* gene cluster from *Sphingomonas* sp. XLDN2-5 and related strains. The *car* genes in the three sphingomonads are more than 99% identity to each other; however, only show 60% identity to that from *Pseudomonas* sp. CA10. (**B**) Comparative analysis of *ant* gene cluster from *Sphingomonas* sp. XLDN2-5 and related strains. The *ant* genes in strain XLDN2-5 and KA1 are more than 99% identity to each other; however, only show 40–76% identity to that from *Burkholderia cepacia* DPO1.

Carbazole-degrading sphingomonads have been isolated in different parts of the world. For example, *Sphingomonas.* sp. GTIN11 was isolated from USA [3], *Novosphingobium* sp. KA1 from Japan [10] and strain XLDN2-5 from China [2]. Although nearly identical *car* and *ant* genes were found in carbazole-degrading sphingomonad strains isolated from geographically dispersed locations, the organization of these genes in strain XLDN2-5 was unique. Figure 5 shows the organization of the known *car* genes (Figure 5A) and *ant* genes (Figure 5B) from different evolutionary origins. There were two truncated ORF1 and ORF8 encoding a putative integrase and a TonB-dependent receptor (Table 1), respectively. ORF1 and ORF8 showed 100% identity to ORF42 and ORF35 in strain KA1 [21], respectively. In strain KA1, ORF42 and ORF35 are intact (Figure 5A), whereas the corresponding ORFs were interrupted by IS*6100* in strain XLDN2-5. Furthermore, BLAST search revealed that the terminal 27-bp region of the reported sequence (1–55 bp in AF442494) in strain GTIN11 is identical to the 27-bp left end of IS*6100*. This suggested that the *carR* gene in strain GTIN11 might be disrupted by IS*6100* element. However, the entire sequence information outside the *car* genes in strain GTIN11 was not reported.

Interestingly, the *ant* cluster in strain XLDN2-5 was also bordered by copies of IS*6100* elements. The organization of *ant* gene cluster was clearly different from that of KA1, in which *catF*, the upstream region of *and* cluster, encodes acetyl-CoA acetyltransferase, while the downstream ORF16 encodes a transposase (Figure 5B). Although there are many ISs belonging to different family, no IS*6100* element was found on pCAR3 [21]. The structure and sequence identified in this study suggested that IS*6100* element might play an important role in the transfer of carbazole-degrading genes. IS*6100* was originally isolated as part of the composite transposon Tn*6100* from *Mycobacterium fortuitum* [16]. Later studies revealed its presence in a wide spectrum of host organisms in distantly related bacterial lineages, e.g. *Sphingomonas paucimobilis* [14], *Arthrobacter* sp. [15], *Pseudomonas aeruginosa* [22], *Xanthomonas campestris* [23], *Salmonella enterica* [24], and *Corynebacterium glutamicum* [25]. The existence of IS*6100* in a very broad host range indicates that the IS*6100* element plays a vital role in disseminating genes, including catabolic and antibiotic resistance genes, among different bacteria. The sequenced copies of IS*6100* from strain XLDN2-5 were 100% identical to those of *Mycobacterium fortuitum*. This supports the hypothesis that IS*6100* elements are distributed widely among microorganisms, whether they are Gram-negative or Gram-positive bacteria [14], [15]. IS*6100* belongs to the IS*6* family of ISs which exclusively integrate through a cointegrative mechanism [13]. The *car* and *ant* genes might become part of strain XLDN2-5 via cointegrative captures. This type of transposition event is consistent with the truncation of the ORF1 and the ORF8 located upstream of *carR* and *carAc*, respectively (Figure 2 and Figure 5A). Thus, the genetic structure of the *car* and *ant* genes in strain XLDN2-5 has shown strong evolutionary trails of gene recruitment and presents an extraordinary example of rearrangements and pathway evolution.

Although the terminal oxygenase and ferredoxin components of CARDO from XLDN2-5 and KA1 are identical, the reductase components have a much lower level of similarity (62%). In general, the genes encoding the components of the known Rieske oxygenases were found closely together in tightly regulated transcriptional units, as was observed with the *car* cluster in strain CA10 (Figure 5A) [26]. However, it is becoming increasingly evident that the genes for catabolic pathways in sphingomonads often locate separately from each other. Recently, it is reported that multiple carbazole degradation genes dispersed on four loci (two *car* loci, one *fdxIfdrI* locus and one *fdrII* locus) on pCAR3 in *Novosphingbium* sp. strain KA1 [10]. The *fdxIfdrI* locus located 50 and 85 kb downstream of *carI* and *carII* gene clusters, while the *fdrII* gene located about 80 and 115 kb downstream of *carI* and *carII* gene clusters. Additional evidence has also been presented for the genes involved in the degradation of PAH by *Sphingobium yanoikuyae* B1 and Q1, and *Novosphingobium aromaticivorans* F199 [11], [12], [27], dibenzo-*p*-dioxin by *Sphingomonas wittichii* RW1 [28], pentachlorophenol by *Sphingmonas chlorophenolica* ATCC 39723 [29], and γ-hexachlorocyclohexane by *Sphingmonas paucimobilis* UT26 [30]. These results suggested that loose association with reductase components might be the characteristic for this type of dioxygenase, which became more independent from the reductase component during evolution.

In the course of cloning *car* genes, we also discovered a novel insertion sequence IS*Ssp1* (see results, IS*6100*::IS*Ssp1*), which insert into the upstream copy of IS*6100*. IS*Ssp1* can be classified as the mutator family that consists of transposases from prokayotes and eukaryotes. There was only one example that an IS could insert into another in *Comamonas* sp. strain JS46 [31]. Thus, it would be interesting to investigate the distribution of IS*Ssp1* in strain XLDN2-5 and its function in the establishment of the carbazole catabolic gene structure.

## Materials and Methods

### Bacterial strains, plasmids and growth conditions

The bacterial strains and plasmids used in this study are listed in Table 2. *Sphingomonas* sp. strain XLDN2-5 utilizes carbazole as the sole carbon and nitrogen source [2], [32]. Strain XLDN2-5 was grown in mineral salt medium (MSM) as previously described [33], and carbazole was added as a 200 mM filter-sterilized stock solution in dimethyl sulfoxide (DMSO). *Escherichia coli* DH5α was used as the recipient strain in all cloning experiments. *E. coli* strains were grown in Luria-Bertani (LB) broth at 37°C. Ampicillin (Amp), when required, was added to a final concentration of 100 µg ml^−1^.

**Table 2:** Bacterial strains and plasmids used.

Strain or plasmid	Description	source
**Bacterial strains**		
*E. coli* DH5α	F- *endA1 hsdR17 supE44 thi-1 recA1 gyrA relA1 Δ(lacZYA-argF) U169 deoR*	TransGen
*Sphingomonas* sp. XLDN2-5	Aerobic, rod shaped, degrades carbazole	Lab stock
**Plasmids**		
pUC19	Amp^r^ *lacZ*, pMB9 replicon, M13IG	TaKaRa
pMD18-T	Clone vector	TaKaRa
pEASY-Blunt	Clone vector	TransGen
pBY13	Amp^r^; pUC19 with 7.8-kb fragment that hybridized with *car* probe	This study
p6100-a	Amp^r^; pMD18-T with 1-kb fragment obtained by genome walking	This study
p6100-1	Amp^r^; pMD18-T with 4.3-kb fragment obtained by IS-based PCR using pIS6100-F1 and pIS6100-R1	This study
p6100-2	Amp^r^; pMD18-T with 2.8-kb fragment obtained by IS-based PCR using pIS6100-F1 and pIS6100-R1	This study
P6100-3	Amp^r^; pMD18-T with 5.2-kb fragment obtained by IS-based PCR using pIS6100-R1	This study
pUcarAa	Amp^r^; pUC19 with 1.2-kb SphI-XbaI fragment containing the *carAa* gene of strainXLDN2-5	This study
pUcarAc	Amp^r^; pUC19 with 0.3-kb XbaI-KpnI fragment containing the *carAc* gene of strain XLDN2-5	This study
pUfdr	Amp^r^; pUC19 with 1.3-kb KpnI-EcoRI fragment containing the *fdr* gene of strain XLDN2-5	This study
pEcarAaAc	Amp^r^; Ka^r^, pEASY-Blunt with 1.5-kb fragment containing the *carAa* and *carAc* genes of strain XLDN2-5	This study
pUcarAaAc	Amp^r^; 1.5-kb SphI-KpnI fragment containing *carAaAc* from pUcarAaAc cloned into SphI-KpnI site of pUC19	This study
pUcarAaAcfdr	Amp^r^; 1.5-kb SphI-KpnI fragment containing *carAaAc* from pUcarAaAc cloned into SphI-KpnI site of pUfdr	This study
pUantAcdb	Amp^r^; pUC19 with 2.1-kb HindIII-EcoRI fragment containing the *antAcAdAb* genes of strainXLDN2-5	This study
pUantAcdba	Amp^r^; pUC19 with 3.5-kb HindIII-EcoRI fragment containing the *antAcAdAbAa* genes of strainXLDN2-5	This study

### DNA manipulation

Total DNA from pure cultures of *Sphingomonas* sp. strain XLDN2-5 was extracted using the Wizard® Genomic DNA Purification Kit according to the recommendations of the manufacturer (Promega Corp., Madison, WI). Restriction endonucleases and T4 DNA ligase were used according to the manufacturer's instructions (TaKaRa). Isolations of DNA fragments from agarose gels were accomplished with the Qiaex II Gel Extraction Kit (Qiagen Corp., Germany). Transformations and agarose gel electrophoresis were carried out using standard methods [34].

### Construction of genomic library

The genomic DNA of *Sphingomonas* sp. XLDN2-5 was mechanically sheared. DNA fragments of 6 to 8 kb were gel purified and used for library construction. The fragments were ligated into pUC19 digested with EcoRV, and dephosphorylated with shrimp alkaline phosphatase (Promega). The library was transformed into electrocompetent *E. coli* DH5α. After incubation at 37°C for 1 h, cells were spread on LB agar plates supplemented with Amp, isopropyl-1-thio-*β*-D-galactopyranoside (IPTG) and 5-bromo-4-chloro-3-indolyl-*β*-D-galactopyranoside. Following overnight incubation at 37°C, plates were scored for white colonies. The white colonies were used for southern blotting using a *car* probe obtained by PCR.

All PCR amplifications were performed with an Eppendorf Authorized Thermal Cycler (Germany) in 50-µl reaction systems containing 5 µl of 10× buffer, 1.5 mM MgCl2, 200 µM dNTPs, 500 pmol of each primer, 10-100 ng of the template DNA, and 2.5 units of DNA polymerase. TransStart FastPfu polymerase (TransGen Biotech Co. Ltd. China) was used in PCRs whose products were used for the construction of plasmids. TaKaRa LA PCR™ Kit Ver. 2.1 was used for IS-based PCR. Taq polymerase (Generay Biotech Co. Ltd. China) was used in other PCRs. Genome walking was performed using a Genome Walking Kit (TaKaRa) according to the manufacturer's protocol. The amplified products obtained by Taq polymerase were gel purified and ligated into vector pMD18-T, followed transformation to competent *E*. *coli* DH5α. All primers used in this study are listed in Table S1.

### Southern blot

Primers pcarF and pcarR were designed according to the *carRAaBaBb* genes of strain KA1 [21]. Products obtained using primers pcarF and pcarR were gel purified and labeled with DIG-11-dUTP, and the DNAs were transferred following the standard protocols [34]. Southern blot was performed using DIG DNA Labeling and Detection Kit (Roche) according to the manufacturer's protocol. Hybridization was performed at 68°C. After hybridization, the membranes were washed twice in a solution containing 2× standard saline citrate (SSC) plus 0.1% sodium dodecyl sulfate (SDS) at 65°C and twice in a solution containing 0.5× SSC plus 0.1% SDS at 68°C.

### DNA sequencing and analysis

Sequencing was performed on an ABI sequencer by Shanghai Invitrogen Biotechnology Co., Ltd, China. The sequences were analyzed with Vector NTI DNA analytical software (version 8). Homology searches were performed with the BLAST programs at the National Center for Biotechnology Information website (http://www.ncbi.nlm.nih.gov/BLAST.html). The deduced amino acid sequences of ORFs were aligned using ClustalW 1.83 [35].

### RNA preparation and RT-PCR

After the precultivation of XLDN2-5 in 5 ml of MSM supplemented with 2 mM carbazole at 30°C, cells were harvested by centrifugation at 5,000 *g* for 5 min and then washed twice using MSM. The washed cells were suspended in 500 ml of MSM. Fifty microliters of the resultant cell suspension was added to 5 ml of MSM supplemented with 5 mM carbazole or with 1 g L^−1^ glucose and 0.5 g L^−1^ NH4Cl. After a 2-h incubation with reciprocal shaking (300 strokes/min) at 30°C, the cells were harvested and used for extraction of total RNA using E. Z. N. A™ Total RNA Kit I (Omega Biotech). Finally, the RNA was eluted in 50 µl of RNase-free water. A PrimeScript™ One Step RNA PCR kit (Takara) was used for RT-PCR, in which 100 ng of total RNA was used as a template. Detailed information on the RT-PCR primer sets and the conditions employed for respective gene amplifications are provided in Table S1. Control experiments without the addition of reverse transcriptase were also included.

### Construction of plasmids for *car* and *ant* genes

The genes, *carAa*, *carAc*, and *fdr* were separately amplified by PCR using the respective primer sets shown in Table S1, which were designed to introduce appropriate restriction sites and the effective ribosome binding sites. In PCR amplification, total DNA of strain XLDN2-5 was used as a template. The amplified products were digested at the introduced restriction sites and ligated into the corresponding sites of pUC19 to produce plasmids (pUcarAa, pUcarAc, and pUfdr) for the expression of single CARDO components. For construction of pEcarAaAc, the *carAa* gene was amplified by carAa-F1 and carAaAcR, and *carAc* gene was amplified by carAaAcF and carAc-R1. *carAa* and *carAc* were then used as templates, while carAa-F1 and carAc-R1 were added to amplify *carAaAc*. The fragment obtained was ligated to pEASY-Blunt to generate pEcarAaAc. After their nucleotide sequences were confirmed to be identical to those designed, pEcarAaAc was double-digested using SphI and KpnI and the 1.5-kb fragment containing *carAaAc* was gel-purified and ligated to the corresponding sites of pUC19 and pUfdr to give pUcarAaAc and pUcarAaAcfdr.

Genes *antAcAdAb* and *antAcAdAbAa* were amplified by PCR using the respective primer sets, which were designed to introduce appropriate restriction sites and the effective ribosome binding sites. The amplified fragments were digested with HindIII and EcoRI before being ligated into pUC19 to generate pUAntAcdb and pUAntAcdba.

### Biotransformation analysis

The *E*. *coli* DH5α strains harboring pUcarAaAcfdr, pUcarAaAc, pUantAcdba or pUantAcdb were cultivated in 5 ml of LB supplemented with Amp at 37°C. Then 100 µl of the culture was transferred to 200 ml of the same medium, and IPTG was added to induce the protein at 37°C for 16 h. Then the cells were harvested by centrifugation (6,000 *g*, 10 min, 4°C), washed twice with MSM, and resuspended in MSM to an OD_600_ of 10. Fifty microliters of carbazole (200 mM in DMSO) or anthranilate (200 mM in DMSO) was added to 10 ml of cell suspensions. After incubation on a reciprocal shaker (200 rpm) at 37°C for 20 h, the mixtures were extracted with an equal volume of ethyl acetate. After derivatization with *N,O*-bis-(trimethylsilyl)trifluoroacetamide (BSTFA) (Sigma) the extracts were analyzed by gas chromatography-mass spectrometry (GC-MS) as described previously [2], [33].

High performance liquid chromatography was carried out to analyze the aqueous samples using an Agilent 1200 series instrument equipped with a variable-wavelength detector and a reversed-phase C18 column (4.6 mm×150 mm, Hewlett-Packard). Residual concentrations of carbazole and anthranilate and the formation of 2′-animobiphenyl-2,3-diol and catechol were determined using a mobile phase of an 80∶20 mixture of methanol and deionized water at a flow rate of 0.5 ml min^−1^.

### Nucleotide sequence accession numbers

The nucleotide sequences of the *car* locus and *ant* locus have been deposited in GenBank under accession numbers GU123624 and GU123625, respectively.

## Supporting Information

Figure S1 IS6100-based PCR. (A) Schematics of relative configurations of two copies of IS6100. The position of primers IS6100-F1, IS6100-F2, IS6100-R1 and IS6100-R2 are shown by arrows. (B and C) Gel electrophoresis of DNA fragments amplified from XLDN2-5 by IS6100-based PCR using IS6100-F1 and IS6100-R1 (lane 1), single IS6100-F1 (lane 2) and single IS6100-R1 (lane 3). Two fragments (4.3 kb and 2.8 kb in size, Figure S1B, lane 1) were amplified using primers IS6100-F1 and IS6100-R1. One specific fragment (5.2 kb in size, Figure S1C, lane 3) using IS6100-R1 was amplified, while no specific fragment was amplified under the same PCR conditions using IS6100-F1 (lane 2). (D) Second-round PCR (lane 4-6) with primer IS6100-F2 using the first-round PCR product as a template.
(1.12 MB TIF)

Figure S2 Determination of the positional relation of TnCar, TnFdr and TnAnt. (A) The positions of primers TnCar-F1, TnFdr-F1, TnFdr-R1, TnAnt-F1, and TnAnt-R1 with blue arrowheads showing their directions. (B) Agarose gel electrophoresis of DNA fragments amplified from the genomic DNA of XLDN2-5 by PCR using TnCar-F1 and TnFdr-R1 (lane 1), TnCar-F1 and TnAnt-R1 (lane 2), TnCar-F1 and TnAnt-F1 (lane 3), TnFdr-F1 and TnAnt-R1 (lane 4), TnFdr-F1 and TnAnt-F1 (lane 5), TnFdr-R1 and TnAnt-R1 (lane 6), and TnFdr-R1 and TnAnt-F1 (lane 7). (C) PCR results using primers carC-sp3 and fdr-r2 (lane 8-12). (D) PCR results (no specific bands) using primers fdr-f2 and antAc-r2 (lane 13-17), and fdr-f2 and antAa-f2 (lane 18-22).
(1.06 MB TIF)

Figure S3 Mass spectra for the products of carbazole transformed by E. coli DH5α harboring pUcarAaAcfdr. GC-MS analysis was performed after trimethylsilylation with BSTFA. Compound I: 2′-aminobiphenyl-2,3-diol; compound II: hydroxycarbazole.
(0.19 MB TIF)

Table S1 Oligonucleotides used in this study for the cloning of genes, genome walking, and the construction of plasmids.
(0.05 MB DOC)
