# Supplementary figures and images for: The Genes Coding for the Conversion of Carbazole to Catechol Are Flanked by IS6100 Elements in Sphingomonas sp. Strain XLDN2-5
Source: PLoS One. 2010 Apr 2;5(4):e10018. doi: 10.1371/journal.pone.0010018 (PMC2848856; doi:10.1371/journal.pone.0010018)

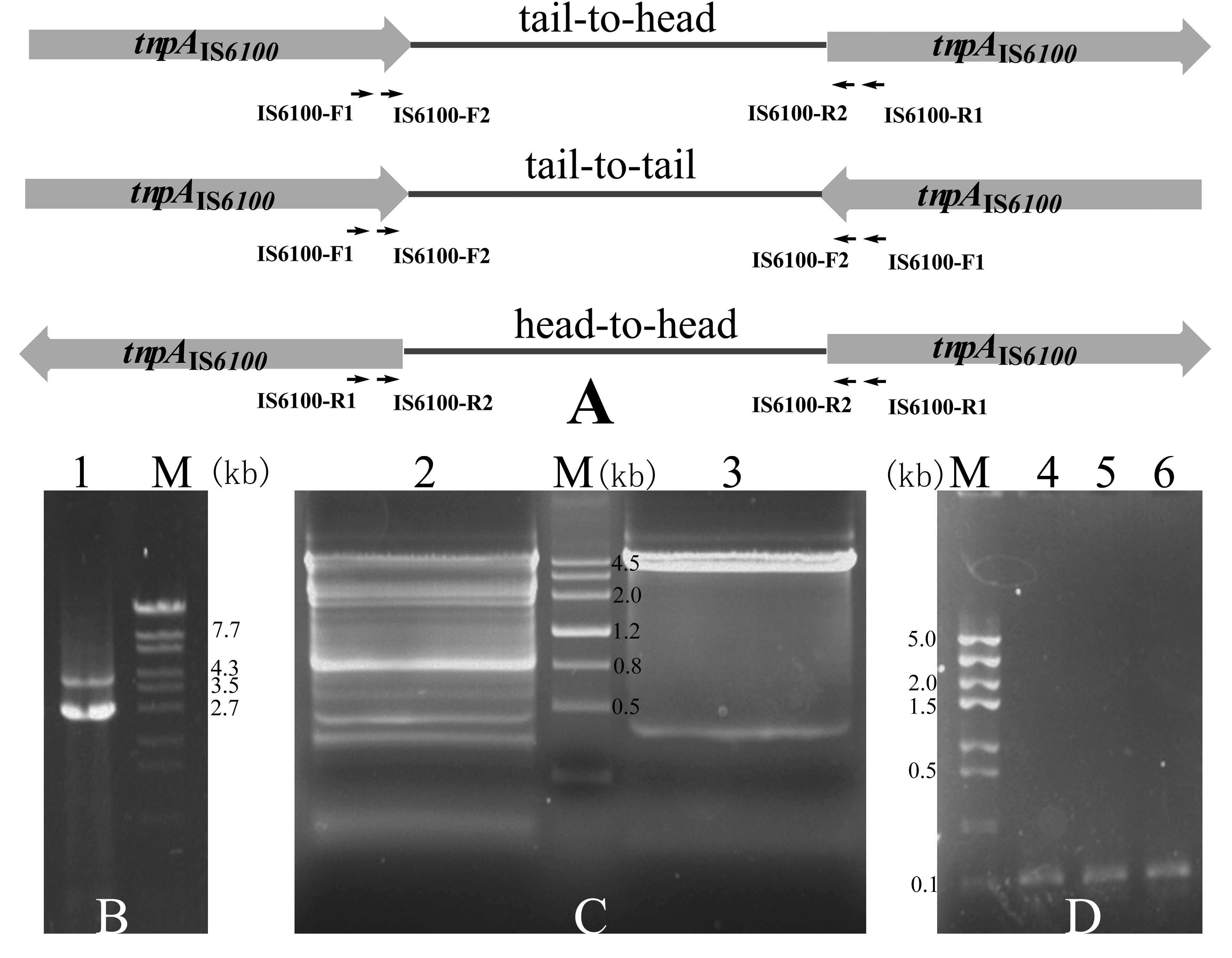

Supplement: Figure S1 — IS6100-based PCR. (A) Schematics of relative configurations of two copies of IS6100. The position of primers IS6100-F1, IS6100-F2, IS6100-R1 and IS6100-R2 are shown by arrows. (B and C) Gel electrophoresis of DNA fragments amplified from XLDN2-5 by IS6100-based PCR using IS6100-F1 and IS6100-R1 (lane 1), single IS6100-F1 (lane 2) and single IS6100-R1 (lane 3). Two fragments (4.3 kb and 2.8 kb in size, Figure S1B, lane 1) were amplified using primers IS6100-F1 and IS6100-R1. One specific fragment (5.2 kb in size, Figure S1C, lane 3) using IS6100-R1 was amplified, while no specific fragment was amplified under the same PCR conditions using IS6100-F1 (lane 2). (D) Second-round PCR (lane 4-6) with primer IS6100-F2 using the first-round PCR product as a template. (1.12 MB TIF) [file pone.0010018.s001.tif]

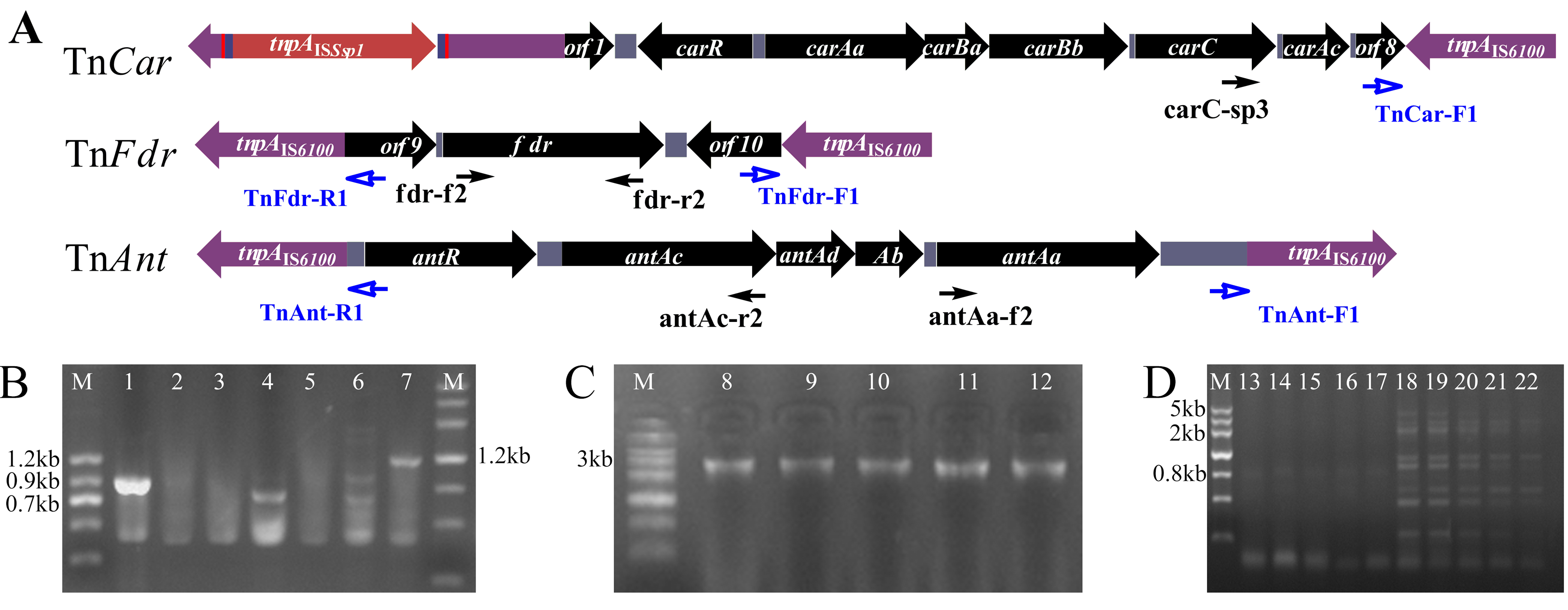

Supplement: Figure S2 — Determination of the positional relation of TnCar, TnFdr and TnAnt. (A) The positions of primers TnCar-F1, TnFdr-F1, TnFdr-R1, TnAnt-F1, and TnAnt-R1 with blue arrowheads showing their directions. (B) Agarose gel electrophoresis of DNA fragments amplified from the genomic DNA of XLDN2-5 by PCR using TnCar-F1 and TnFdr-R1 (lane 1), TnCar-F1 and TnAnt-R1 (lane 2), TnCar-F1 and TnAnt-F1 (lane 3), TnFdr-F1 and TnAnt-R1 (lane 4), TnFdr-F1 and TnAnt-F1 (lane 5), TnFdr-R1 and TnAnt-R1 (lane 6), and TnFdr-R1 and TnAnt-F1 (lane 7). (C) PCR results using primers carC-sp3 and fdr-r2 (lane 8-12). (D) PCR results (no specific bands) using primers fdr-f2 and antAc-r2 (lane 13-17), and fdr-f2 and antAa-f2 (lane 18-22). (1.06 MB TIF) [file pone.0010018.s002.tif]

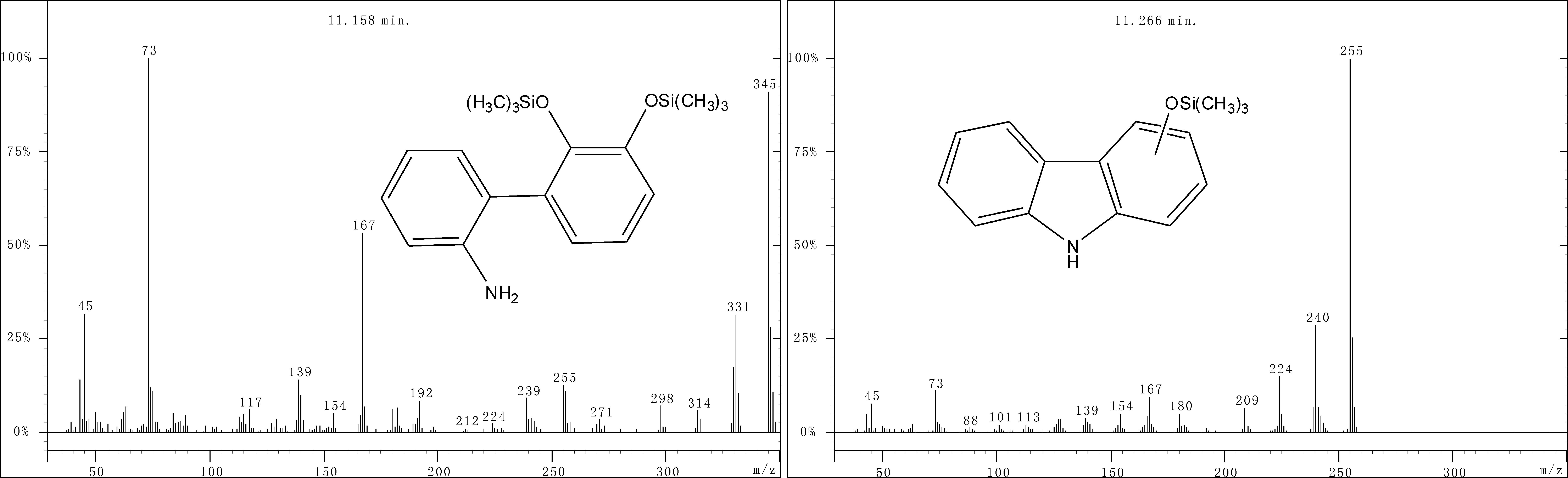

Supplement: Figure S3 — Mass spectra for the products of carbazole transformed by E. coli DH5α harboring pUcarAaAcfdr. GC-MS analysis was performed after trimethylsilylation with BSTFA. Compound I: 2′-aminobiphenyl-2,3-diol; compound II: hydroxycarbazole. (0.19 MB TIF) [file pone.0010018.s003.tif]
