# Supplementary material for: The Genes Coding for the Conversion of Carbazole to Catechol Are Flanked by IS6100 Elements in Sphingomonas sp. Strain XLDN2-5
Source: PLoS One. 2010 Apr 2;5(4):e10018. doi: 10.1371/journal.pone.0010018 (PMC2848856; doi:10.1371/journal.pone.0010018)
Supplement: Table S1 — Oligonucleotides used in this study for the cloning of genes, genome walking, and the construction of plasmids. (0.05 MB DOC) [file pone.0010018.s004.doc]

Table S1. Oligonucleotides used in this study for the cloning of genes, genome walking, and the construction of plasmids

| Primer | Sequence | Target gene | PCR condition |
| --- | --- | --- | --- |
| **For Southern hybridization** | | | |
| pcarF  pcarR | 5'-TTCTCCTAGCCACCCACAA-3'  5'-GCCATGAACCAGCACCAC-3' | *carAaBaBbC* | 30 s 95°C; 30 s 65°C; 2.5 min 72°C; 30 cycles |
| **For genome walking** | | |  |
| carAc-sp1  carAc-sp2  carAc-sp3 | 5'-CGCAAAGGTCCGCGTGATCTTC-3'  5'-GTGGACGGTATCGAAGCCGTTT-3'  5'-GAAATGCTCGATTGCGTAGCGG-3' | *carAc*  *carAc*  *carAc* | According to the manufacturer’s instruction |
| **For Insertion Squence-Based PCR** | | | |
| IS6100-F1  IS6100-F2  IS6100-R1  IS6100-R2 | 5'-ATGCGAGCCCTGCGCAAAGGAC-3'  5'-GCAGAGCGACAGCCTACCTCTGACTG-3'  5'-GCCAGCATTTCCTCAAGGTCGC-3'  5'-TCCGCCAAGCATGCTCAAGCTTCACG-3' | IS*6100*  IS*6100* | 30 s 95ºC; 30 s 63ºC; 7 min 72ºC; 30 cycles |
| **For analysis the relation between Tn*Car* Tn*Fdr* and Tn*And*** | | | |
| TnCar-F1  TnFdr-F1  TnFdr-R1  TnAnt-F1  TnAnt-R1  carC-sp3 | 5'-ATCAACAATGTGTCGGACGGCTCTG-3'  5'-CATCGCGCAGGACAGCTCCTC-3'  5'-CGAACTGGTAGGCGCCCTGG-3'  5'-CGCATATTGGAGATGCCGGGC-3'  5'-CACGTCAGACCCGCTCGACG-3'  5'-AAGGAGGTGACGCAATGACCGC-3' | Tn*Car*  Tn*Fdr*  TnFd*r*  Tn*Ant*  Tn*Ant*  Tn*Car* | 30 s 95ºC; 30 s 65ºC; 1.2 min 72ºC; 30 cycles |
| **For RT-PCR** | | | |
| carAaBaBb-f1  carAaBaBb-r1 | 5'-TTCCCGGTTGAAGAGGCCAAAGG-3'  5'-GGTCCGAGCCGATGACGATCAGC-3' | *carAaBaBb* | 30 min 50ºC; 2 min 94ºC; 30 s 94ºC; 30 s 65.2ºC; 1.2 min 72ºC; 30 cycles |
| carBbC-f1  carBbC-r1 | 5'-TGTTCATCGACCCCGATGGCAGG-3'  5'-CCACCCATCGAATTGCCGACAAG-3' | *carBbC* | 30 min 50ºC; 2 min 94ºC; 30 s 94ºC; 30 s 63.6ºC; 0.8 min 72ºC; 30 cycles |
| carCAc-f1  carCAc-r1 | 5'-CTCGTCCGTGCACTAACGACCG-3'  5'-CCGCTACGCAATCGAGCATTTC-3' | *carCAc* | 30 min 50ºC; 2 min 94ºC; 30 s 94ºC; 30 s 65.2ºC; 0.6 min 72ºC; 30 cycles |
| fdr-f2  fdr-r2  antAcAdAb-f1  antAcAdAb-r1  antAa-f1  antAa-r1 | 5'-CCCGCTCTCCAAGGAGTATCTGG-3'  5'-TTGGTGGCGTTGACGCAGTCGAG-3'  5'-TGCCCTATGCTGTCTTCAGTTCC-3'  5'-CTTGGGTTCGCCGGTGGTGA-3'  5'-GAGGGTTATGACGGCCGCATC-3'  5'-CTTGTCCTGTTCCAGCGCCTTG-3' | *fdr*  *antAcAdAb*  *antAa* | 30 min 50ºC; 2 min 94ºC; 30 s 94ºC; 30 s 63.6ºC; 1.0 min 72ºC; 30 cycles  30 min 50ºC; 2 min 94ºC; 30 s 94ºC; 30 s 63 ºC; 1.0 min 72ºC; 30 cycles |
| **For construction plasmids** | | | |
| carAa-F1  carAa-R1 | 5'-AAAAGCATGCCT*AAGGAGG*TGTTCATatgGCTAACCAACCATCAATCG-3'  5'-AAAATCTAGAtcaACGTGCTTCCTGAATGCCGCG-3' | *carAa* | 30 s 95ºC; 30 s 65ºC; 1.2 min 72ºC; 30 cycles |
| carAc-F1  carAc-R1 | 5'-AAAATCTAGACT*AAGGAGG*TGTTCATatgACCGCAAAGGTCCGCGTGATC-3'  5'-AAAAGGTACCtcaTGACTGTGCCTTCGGCAGTTC-3' | *carAc* | 30 s 95ºC; 30 s 65ºC; 0.3 min 72ºC; 30 cycles |
| fdr-F1  fdr-R1 | 5'-AAAAGGTACCCT*AAGGAGG*TGTTCATatgACTGATACGCACTATGACGTC-3'  5'-AAAAGAATTCtcaAGCAGGGCTTGGCGTCATGAAG-3' | *fdr* | 30 s 95ºC; 30 s 65ºC; 1.3 min 72ºC; 30 cycles |
| carAaAcF  carAaAcR | 5'-GGAAGCACGTtgaTGGGCAC*AAGGAGG*TGACGCAatgACCGCAAAG-3'  5'-CTTTGCGGTcatTGCGTCACCTCCTTGTGCCCAtcaACGTGCTTCC-3' | *carAaAc* | 30 s 94ºC; 30 s 45ºC; 1.5 min 72ºC; 5 cycles; 30 s 94ºC; 30 s 65ºC; 1.5 min 72 ºC; 30 cycles |
| antAc-F3  antAb-R3  antAa-R4 | 5'-ACTGAAGCTTCGAAGGAGGTGCTATCatgATCACTGAGAAAACGG-3'  5'-GACTGAATTCtcaAAGTTCCACCTCGATCCGGTC-3'  5'-GACTGAATTCCGAAATGCAGCCCATCTtcaGG-3' | *antAcAdAb*  *antAcAdAbAa* | 30 s 95ºC; 30 s 62ºC; 4 min 72ºC; 30 cycles |
